# Adherence to the Caffeine Intake Guideline during Pregnancy and Birth Outcomes: A Prospective Cohort Study

**DOI:** 10.3390/nu10030319

**Published:** 2018-03-07

**Authors:** Amy Peacock, Delyse Hutchinson, Judy Wilson, Clare McCormack, Raimondo Bruno, Craig A. Olsson, Steve Allsop, Elizabeth Elliott, Lucinda Burns, Richard P. Mattick

**Affiliations:** 1 National Drug and Alcohol Research Centre, University of New South Wales, Sydney, NSW 2052, Australia; delyse.hutchinson@deakin.edu.au (D.H.); JudyWilson@live.com.au (J.W.); clare.a.mccormack@gmail.com (C.M.); Raimondo.Bruno@utas.edu.au (R.B.); l.burns@unsw.edu.au (L.B.); R.Mattick@unsw.edu.au (R.P.M.); 2 School of Medicine (Psychology), University of Tasmania, Hobart, TAS 7001, Australia; 3 Centre for Social and Early Emotional Development, School of Psychology, Faculty of Health, Deakin University, Melbourne, VIC 3125, Australia; craig.olsson@deakin.edu.au; 4 Murdoch Children’s Research Institute, Centre for Adolescent Health, Royal Children’s Hospital, Melbourne, VIC 3052, Australia; 5 Department of Paediatrics, Royal Children’s Hospital, Melbourne University, Melbourne, VIC 3052, Australia; 6 Department of Psychiatry, Columbia University Medical Center, New York, NY 10032, USA; 7 National Drug Research Institute, Curtin University, Perth, WA 6845, Australia; S.Allsop@curtin.edu.au; 8 Discipline of Child and Adolescent Health, The University of Sydney, The Children’s Hospital at Westmead, Sydney, NSW 2145, Australia; elizabeth.elliott@health.nsw.gov.au

**Keywords:** pregnancy, birth outcomes, birth weight, caffeine, coffee, small for gestational age

## Abstract

The aims of this study were to identify: (i) the proportion of women exceeding the caffeine intake guideline (>200 mg/day) during each trimester, accounting for point of pregnancy awareness; (ii) guideline adherence trajectories across pregnancy; (iii) maternal characteristics associated with trajectories; and (iv) association between adherence and growth restriction birth outcomes. Typical and maximal intake per consumption day for the first trimester (T1; pre- and post-pregnancy awareness), second (T2), and third trimester (T3) were recorded for a prospective cohort of pregnant Australian women with singleton births (*n* = 1232). Birth outcomes were birth weight, small for gestational age, and head circumference. For each period, participants were classified as abstinent, within (≤200 mg), or in excess (>200 mg). Latent class growth analyses identified guideline adherence trajectories; regression analyses identified associations between adherence in each trimester and birth outcomes. The percentage of participants who reported caffeine use declined between T1 pre- and post-pregnancy awareness (89% to 68%), and increased in T2 and T3 (79% and 80%). Trajectories were: ‘*low consumption*’ (22%): low probability of any use; ‘*within-guideline*’ (70%): high probability of guideline adherence; and ‘*decreasing heavy use*’ (8%): decreasing probability of excess use. The latter two groups were more likely to report alcohol and tobacco use, and less likely to report planning pregnancy and fertility problems. Exceeding the guideline T1 pre-pregnancy awareness was associated with lower birth weight after covariate control (b = −143.16, *p* = 0.011). Overall, high caffeine intake pre-pregnancy awareness occurs amongst a significant minority of women, and continued excess use post-pregnancy awareness is more common where pregnancy is unplanned. Excess caffeine consumption pre-pregnancy awareness may increase the risk for lower birth weight. Increasing awareness of the guideline in pregnancy and preconception health care may be warranted.

## 1. Introduction

Caffeine (1,3,7-trimethylxanthine) is one of the most commonly consumed substances worldwide, available in coffee, tea, soft drinks, chocolate, and many other products [1]. Given the pervasiveness of its use and availability, it is not surprising that most women report caffeine use throughout pregnancy (e.g., [2,3]). However, potential adverse effects in regard to birth, childhood, and later life outcomes have generated concern [4,5]. Orally-ingested caffeine is rapidly absorbed, reaching peak plasma concentration between 30–45 min with a plasma half-life of 5 to 6 h [6]. Caffeine passes through the placenta and blood-brain barrier and caffeine metabolites accumulate in the foetal brain [7]. Caffeine exposure can cause uteroplacental vasoconstriction and foetal hypoxia [8]. The half-life of caffeine increases between the first and third trimester, resulting in increased foetal exposure [9].

Systematic reviews have highlighted a dose-response relationship between caffeine intake and poorer birth outcomes, including lower birth weight and a greater likelihood of miscarriage and stillbirth [4,10,11]. Various organisations have established guidelines recommending caffeine consumption of no more than 200 mg/day (equivalent to approximately two ground coffees) for women who are pregnant [12,13,14,15,16]. The rationale for this upper-limit is often not specified, however, and could best be described as a precautionary principle. Evidence cited by the American College of Obstetricians and Gynaecologists include a small number of prospective cohort studies indicating that for women consuming up to 200 mg/day, risk of miscarriage [17,18], and preterm birth [19,20] was similar to the risk for those consuming no caffeine. 

Several studies that document caffeine intake within each trimester of pregnancy indicate that use may be reduced relative to pre-pregnancy levels, and is typically lower during trimester 1 (T1) and 2 (T2) than trimester 3 (T3) (e.g., [2,3,21]). However, there is limited epidemiological evidence available regarding use across trimesters with respect to adherence to the guideline of ≤200 mg/day. Further to this, caffeine intake is often averaged for T1 and/or across pregnancy despite evidence of altered substance use with pregnancy awareness, as described in this cohort [22] and in others [23], with respect to alcohol use. As such, our aims were to describe:

1. Caffeine use in each trimester, taking into account point of pregnancy awareness;
2. Guideline adherence trajectories (abstinent, within the guideline, exceeding the guideline) across pregnancy;
3. Maternal characteristics associated with guideline adherence trajectories; and
4. Associations between exceeding the guideline (within each trimester and across pregnancy) with growth restriction at birth.

## 2. Materials and Methods 

### 2.1. Participants and Procedure

The Triple B (‘*Bumps, Babies, and Beyond*’) Pregnancy Cohort Study comprises a prospective cohort of pregnant women and their partners recruited in 2009–2013 through public hospital antenatal clinics in New South Wales (NSW; *n* = 1305) and Western Australia (WA; *n* = 318). Participating hospitals in NSW were the Royal Prince Alfred Hospital, Camperdown; The Royal Women’s Hospital, Randwick; and Liverpool Hospital, Liverpool. Participants in Western Australia were recruited through the King Edward Memorial Hospital, Subiaco. Sites were chosen because they had attached specialist antenatal services for women affected by substance-related problems in pregnancy. Pregnant women were consecutively approached and invited to participate in the study by research officers who attended antenatal clinics at each hospital, across all days and months of the year to represent (proportionally) all clinics operating at each recruitment site. Eligibility criteria comprised: being pregnant; ≥15 years of age; no major medical complications (mother or foetus); intention of mother or both parents to be the primary caregiver/s; being mentally able to provide consent and complete assessments; and sufficient literacy in English. Interviews were conducted by telephone or in person by trained interviewers. Infant data were collected at the eight-week postnatal follow-up. Ethics approval was granted by the Sydney South West Area Health Service Human Research Ethics Committee and the University of New South Wales Human Research Ethics Committee (reference: HC08224; 29/08/2008), and all participants gave informed consent. Full details of the study protocol are available in Hutchinson et al. [24]. 

### 2.2. Measures

#### 2.2.1. Exposure Variables

##### Maternal Caffeine Use

Participants were asked to indicate caffeine use in each trimester. Consumers were asked to specify the type and quantity of caffeinated beverages consumed in terms of typical and maximal intake, and frequency (‘everyday’, ‘5–6 times a week’, ‘3–4 times a week’, ‘1–2 times a week’, ‘2–3 times a month’, ‘once a month’, ‘once or twice during the period’). Project staff subsequently calculated approximate milligrams consumed by each participant based on the caffeine content reported by manufacturers or by the Australian Institute of Sport [25]. In T1 interviews, participants were asked to specify the time of pregnancy awareness, and to identify caffeine use from estimated time of conception to their point of pregnancy awareness (labelled ‘*T1 pre-pregnancy awareness*’). They were then asked about intake post-awareness to the end of T1 (labelled ‘*T1 post-pregnancy awareness*’), and in T2 and T3. Participants were then categorized into three levels of caffeine consumption within T1 pre-pregnancy awareness, T1 post-pregnancy awareness, T2, and T3 periods: (i) no caffeine use; (ii) typical or maximal intake on consumption days within the intake guideline (≤200 mg); and, (iii) typical or maximal intake on consumption days exceeding the intake guideline (>200 mg). We opted to categorise participants by typical or maximal intake to capture chronic and acute high exposure, respectively. Frequency of use was dichotomized into ≥3 days a week versus <3 days a week.

#### 2.2.2. Outcome Variables

##### Infant Characteristics/Birth Outcomes

In addition to infant sex, birth outcomes included: (i) body weight: weight in grams at birth; (ii) small-for-gestational age (SGA): birth weight less than the 10th percentile for gestational age per Australian norms [26]; and, (iii) head circumference (centimetres). Birth outcomes were extracted at eight-weeks from hospital data recorded in their *Blue Book*, a national infant health record [27]. 

#### 2.2.3. Potential Confounding Variables

##### Socio-Demographics

Maternal age at birth was calculated by subtracting the mother’s date of birth from infant’s date of birth. During the first interview (T1), participants also reported their country of birth, current employment status, highest education qualification, and whether they were a single parent. The Index of Relative Socio-Economic Advantage and Disadvantage (IRSAD) from the Socio-Economic Indexes for Areas (SEIFA) data package was used to classify participants into low, moderate, or high socio-economic status (SES) based on residential postcode [28]. 

##### Characteristics of Pregnancy

In the T1 interview, participants were asked how they felt about falling pregnant (‘I wanted to become pregnant’, ‘I didn’t want to become pregnant’, ‘I hadn’t thought about becoming pregnant’, ‘other’). Responses were coded as pregnancy ‘actively planned’ versus ‘not actively planned’; ‘other’ responses (*n* = 69) were classified based on examination of open-ended responses. Parity (total number of pregnancies carried to term) was coded as ‘0’ or ‘≥1 full-term pregnancies’. Fertility problems were assessed by asking participants whether they required any assistance/fertility treatment in becoming pregnant (yes/no).

##### Substance Use and Health

Participants reported whether they had used alcohol, tobacco, or illicit drugs within each trimester, and any use of each during pregnancy was coded. In T1, participants were administered the Anxiety and Stress subscales of the Depression Anxiety Stress Scale [29]; scores of >10 and >19 at any point during pregnancy were considered indicative of elevated anxiety and stress, respectively. The self-report Edinburgh Postnatal Depression Scale [30] was administered at T1 to measure depression; scores >9 were considered elevated. This tool is validated for the assessment of women who are currently pregnant (e.g., [31]). Pre-pregnancy body mass index (BMI) was calculated based on the mothers’ retrospectively self-reported pre-pregnancy weight and height [32]. 

### 2.3. Data Analysis

Latent class growth models (1–5 classes) were constructed based on whether participants reported abstinence, typical/maximal use never exceeding the guideline (≤200 mg), or typical/maximal use exceeding the guideline (>200 mg) in T1 pre-pregnancy awareness, T1 post-pregnancy awareness, T2 and T3; the fit of each model was compared using MPlus v7 (Los Angeles, CA, USA) [33]. Non-equidistant time scores for the slope growth model comprised the following week intervals: 5 (median point of pregnancy awareness), 12 (end of T1), 27 (end of T2), and 40 (median gestation and, thus, end of T3). Results of the 5-class model are not presented because results suggested that the model was under-identified. 

Three criteria were used for model selection [34]. Akaike’s information criterion (AIC) and sample-size adjusted Bayesian information criterion (ssaBIC) were used to assess model fit; lower values indicated better fit. The Lo-Mendell-Rubin adjusted log-likelihood ratio test (LMR-ALRT) statistic [35] was used to compare fit of a *k* class model with a *k*-1 class model; *p* < 0.05 indicated that the latter model should be rejected for the former. Entropy indexed class classification accuracy; higher values (range 0.0–1.0) indicated better differentiation of individuals. Class composition of models was examined alongside fit statistics to determine the most parsimonious and meaningful class structure. Baseline covariates were not included as it was our intent to control for these factors in multivariate analyses for birth outcomes (see below). Sensitivity analyses were conducted whereby models were estimated using a standardised division of T1 into two halves with the end of the sixth week used as the midpoint, to ensure that results were not unduly influenced by individual variation by point of pregnancy awareness [36].

The most likely trajectory group for each participant based on the chosen model was used in subsequent analyses. The associations between trajectory group and socio-demographic factors, substance use, and birth outcomes were analysed using multinomial logistic regression. Descriptive statistics comprised percentages for categorical data and the median and interquartile range (IQR) for continuous data (mother’s age at birth and pre-pregnancy BMI) due to significant skew. 

A series of linear regression models (dependent variables: birth weight, head circumference) and logistic regression models (dependent variable: SGA) were used to examine the association between guideline adherence and birth outcomes. Analyses were conducted in Stata v14 (StataCorp LP, College Station, TX, USA) [37] using robust methods. Separate models were run using caffeine use trajectory group and T1 pre-pregnancy awareness, T1 post-pregnancy awareness, T2 and T3 use guideline adherence (abstinent, typical/maximal intake never exceeds guideline, typical/maximal intake exceeding the guideline) in the first step of the model. Maternal socio-demographic variables (age at birth, education, SES, country of birth and single parent household) and infant variables (sex) associated with birth outcomes in prior research were included in the second step [38,39,40]. Variables significantly associated with caffeine trajectory group in multinomial logistic regression were included in the third step. These analyses identified the strength of association with birth outcomes when describing trajectories of guideline adherence across pregnancy versus within each stage of pregnancy. Across all analyses, significance levels were maintained at *p* < 0.050. 

## 3. Results

### 3.1. Participants

Of the women approached to participate (*n* = 6167), 4266 met the eligibility criteria and 1634 (38.3%) consented and completed at least one study measure. These recruitment rates are consistent with other recent longitudinal cohorts with hospital-based recruitment and with a high degree of participant commitment [41]. Of these, 1456 women (89.1%) completed all four interviews: T1 (conception to 12-weeks), T2 (13-weeks to 27-weeks), T3 (28-weeks to birth), and eight-week follow-up (eight-weeks postnatal). Of the 1456 mothers who completed all four requisite interviews, 1232 were retained (see Table S1). 

### 3.2. Maternal and Infant Characteristics

The median maternal age at birth was 33 years (IQR 29–36). As recorded in T1, over half of participants were born in Australia (56%), were of a high SES (64%), in paid employment (68%), held a tertiary qualification (66%), and lived with their partner (93%). Participants were also typically nulliparous (57%) and had planned their pregnancy (78%), with 8% experiencing fertility problems. 

The infant sample comprised a near even-split of males (52%, *n* = 637) and females (48%, *n* = 595). One tenth (10%, *n* = 125) of infants were classified as SGA; median birth weight was 3430 grams (IQR 3131–3760); median weeks gestation was 39.5 (IQR 38.5–40.2); and median head circumference was 35 centimetres (IQR 34–35.5).

### 3.3. Caffeine Consumption

The percentage of women reporting any caffeine use and median typical intake amongst consumers decreased from pre- to post-pregnancy awareness in T1 (89% versus 68%; 107 mg versus 60 mg, respectively). Rates of use and median typical intake increased, but did not return to pre-awareness levels, in T2 and T3. Four-fifths used caffeine regularly (i.e., three or more days per week) across each trimester (Table 1). The percentage exceeding the guideline (>200 mg) on typical/maximal consumption days over trimesters declined and then plateaued: 30% reported typical/maximal intake exceeding the guideline T1 pre-pregnancy awareness; 10% T1 post-pregnancy awareness; and 4% and 5% in T2 and T3, respectively.

### 3.4. Guideline Adherence Trajectories

Fit statistics for the 1- to 4-class model showed that AIC and ssaBIC were smallest and entropy was greatest for the 4-class model (Table 2). However, the chi-square test did not indicate improved model fit for the 4- over a 3-class solution, whilst the 3-class solution was a significant improvement on the 2-class solution. Examination of class composition supported selection of the 3-class model: each class was substantive and distinct in their consumption patterns across pregnancy (Figure S1). Sensitivity analyses with a standardized division of T1 (sixth week used as midpoint) showed comparable fit statistics and classification (Figure S1); thus, the original 3-class model was retained.

One-fifth of mothers (22%, *n* = 226) had a low probability of reporting typical/maximal caffeine consumption exceeding (<0.01) or within the guideline (<0.32) within any trimester (thus labelled ‘*low consumption group*’). Over two-thirds (70%, *n* = 866) had a low probability (<0.12) of reporting typical/maximal caffeine consumption exceeding the guideline at any point during pregnancy, and a high probability (~0.82) of reporting use which did not exceed the guideline across trimesters (‘*within guideline group*’). The final class, comprising one-tenth of the sample (8%, *n* = 100), had a high proportion of consumers (0.82) reporting typical/maximal caffeine intake exceeding the guideline at T1. Across trimesters, the number reporting these consumption practices declined: by T3, this group had a similar probability of being within the guideline (0.48) versus exceeding the guideline (0.51) (thus labelled ‘*decreasing heavy use group*’).

### 3.5. Correlates of Guideline Adherence Trajectory

The ‘*low consumption group*’ was the referent category for multinomial logistic regression models (Table 3). Analyses showed that the ‘*within guideline group*’ and the ‘*decreasing heavy use group*’ had a greater relative risk ratio (RRR) of having carried a child to full-term previously, and a lower RRR of being born in a non-English speaking country, holding a tertiary qualification, planning their pregnancy, and having fertility problems, than the ‘*low consumption group*’. Whilst both groups had a greater RRR of alcohol use and tobacco use during pregnancy relative to the ‘*low consumption group*’, the ‘*decreasing heavy use group*’ also had greater RRR of reporting illicit drug use during pregnancy, as well as a higher pre-pregnancy BMI. No statistically significant difference was observed between the groups in age, SES, employment, education, and the percentage who were living with their partner.

### 3.6. Association between Guideline Adherence and Birth Outcomes

Relative to caffeine abstinence, exceeding the guideline in T1 pre-pregnancy awareness was associated with a significant decrease, and exceeding the guideline in T3 was associated with a significant increase, in birth weight after controlling for potential confounders (Table 4). Whilst birth weight tended to be lower for those who reported exceeding the guideline T1 post-pregnancy awareness, and for those whose trajectory of use over pregnancy was described as ‘decreasing heavy use’, the association did not reach statistical significance after controlling for confounders (see Table S2 for descriptive statistics). 

Neither trajectory group nor guideline adherence at any time point was significantly associated with SGA in unadjusted and adjusted logistic regression models (Table 4). Similarly, neither trajectory group nor guideline adherence at each of the four time points was significantly associated with head circumference in unadjusted and adjusted linear regression models (Table 4).

## 4. Discussion

This study comprised a unique approach to assessing caffeine use in pregnancy, distinct by differentiating use pre- and post-pregnancy awareness, and identifying the trajectory of use across pregnancy (as well as within each trimester, as per existing research; [10,11]). Previous research has shown that caffeine use during pregnancy declines from T1 to T2, increasing thereafter [2]. However, the current study indicated a change in caffeine use earlier than T2, occurring around the point of pregnancy awareness. As most women become aware of pregnancy in T1, a change in health behaviours around this time might be expected. The current findings are, thus, important from an epidemiologic perspective, demonstrating heterogeneity in caffeine use between and within trimesters, and from a clinical perspective, demonstrating the importance of emphasizing engagement in preconception health behaviours.

The trajectories of caffeine guideline adherence identified in the current study indicate substantial variation in use across pregnancy. One-fifth typically reported abstinence throughout pregnancy (‘*low Consumption group*’); two-thirds continued consumption, but in adherence with the guideline, throughout pregnancy (‘*within guideline group*’); and one-tenth typically exceeded the guideline across pregnancy (‘*decreasing heavy use group*’). These findings suggest that treating this population as a homogeneous group across pregnancy will reduce sensitivity. It is important to note that the majority of women did not report guideline adherence throughout pregnancy, which may indicate general awareness of the intake recommendation. Notably, median typical and maximum intake for the sample at each time point did not exceed the guideline. However, the subgroup engaged in the heaviest use (‘*decreasing heavy use group*’) displayed the most distinct shift in use across pregnancy: four-fifths reported exceeding the guideline in T1 pre-pregnancy awareness, while only half did so in T3. Those who altered their excess use generally transitioned to use in adherence with the guideline as opposed to abstinence. 

Unique profiles associated with guideline adherence trajectories reflect existing evidence showing that heavy caffeine use during pregnancy is associated with lower educational attainment, history of full-term childbirth, and alcohol and tobacco consumption throughout pregnancy [42,43]. The ‘*within guideline group*’ and ‘*decreasing heavy use group*’ were also more likely to report any tobacco and alcohol use during pregnancy, and the latter group were more likely to report illicit drug use during pregnancy, as well as greater pre-pregnancy BMI. Potential co-occurrence of high-risk behaviours is concerning given the consequences for foetal growth outcomes [44,45], and suggests that maternal caffeine use should be considered in future research investigating effects of alcohol and tobacco on infant development.

In regard to birth outcomes, exceeding the guideline T1 pre-pregnancy awareness was associated with a significant reduction in birth weight after controlling for confounders. The magnitude of this effect (143 g) was greater than evident when looking at the association between caffeine use within each trimester and birth weight outcomes in previous research (~60–80 g) [2,3,43]. The observation that this association was restricted to the T1 pre-pregnancy awareness period reinforces the need for a precautionary approach in regard to caffeine and other substance use in preconception health care, particularly as those who report exceeding the guideline in this period are at higher risk of alcohol, tobacco, and illicit drug use during pregnancy. This is particularly important given health professionals’ poor understanding of caffeine pharmacokinetics and their poor awareness of the caffeine intake guideline [46]. 

While these results have significant public health and clinical implications, it should be noted that the magnitude of the effect in the current study (and the existing literature) is modest relative to other risk factors, such as smoking [4]. Further, in contrast with expectations, exceeding the guideline in T3 was associated with an increase in birth weight, highlighting the importance of considering both dose and timing in regards to caffeine use throughout pregnancy. Exceeding the guideline across pregnancy and within each trimester was neither associated with SGA nor head circumference after controlling for confounders. Meta-analyses have shown a dose-response relationship between caffeine intake and SGA [4,10], although, as aforementioned, effect sizes are small. Greater representation from heavier caffeine consumers (e.g., >300 mg/day) and issues around unmeasured or poorly assessed confounding factors in these studies should also be noted.

The Triple B cohort provides the most comprehensive longitudinal assessment of substance use in the perinatal period to date in Australia, allowing for complex modelling of trajectories of use across pregnancy. Power analyses for the hierarchical linear regression models indicated that a sample of 1032 would be required to detect an effect as small as 1% *R*^2^ by the tested variable controlling for the fourteen covariates that, themselves, explain 1%. This suggests that the current sample (*n* = 1232) provided sufficient power to detect meaningful differences, although residual confounding should be noted as a possible limitation. However, the study design may have implications for the generalizability of findings. A comparison with general population estimates suggest the Triple B cohort was similar to the general population in rates of employment and the proportion of Aboriginal or Torres Strait Islander origin. However, cohort participants were slightly older, more socio-economically advantaged, more likely to be born overseas, more highly educated, more likely to be nulliparous, and less likely to be living in single-parent households. The sex distribution of infants in the study was also similar to Australian population figures although other infant characteristics differed from the general population, with gestational age at birth, birthweight, and the proportion of twins/multiple births being slightly higher in the cohort [24]. Critical to birth weight, this study comprised only singleton births, and excluded multiple births and pregnancy resulting in miscarriage. No subjective measurement of caffeine intake from products other than beverages were included. These products (with the exception of pure caffeine products, such as caffeine tablets or sprays) typically contain low quantities of caffeine, yet quantification of their intake is important when assessing maximal and acute intake. Participants were not asked about the mode of preparation for tea or coffee, with evidence that caffeine content varies by grinding and brewing technique [47]. Objective measurement of caffeine metabolites, changes in caffeine metabolism across pregnancy, and the limitations of retrospective self-report of caffeine use [48] are common challenges in measuring caffeine and other drug use. However, evidence points to maternal self-report of intake as the optimal measurement approach for caffeine exposure [48], and the large sample size in the present study in the context of existing Australian research should be noted as a strength in this respect. 

## 5. Conclusions

The majority of the cohort reported using caffeine on a frequent basis throughout pregnancy. Distinct trajectories of guideline adherence were observed with a substantial, yet declining, proportion of women reporting exceeding the guideline as pregnancy progressed. There was a shift in caffeine use around the self-reported point of pregnancy awareness. Further, an association between exceeding the guideline pre-pregnancy awareness T1 and lower birth weight highlights the importance of this early period in foetal development. Considerable debate still exists regarding the causal role of caffeine intake in adverse birth outcomes, although biological plausibility, mounting evidence from observational studies, and suggestions of a dose-dependent relationship warrant a precautionary stance in pregnancy and pre-conception healthcare. Indeed, these findings highlight the potential need for targeted education and intervention strategies aimed at reducing prenatal caffeine intake among higher-risk women. From an epidemiologic perspective, these findings highlight the need to consider heterogenity in use within/between trimesters and across individuals to ensure sensitivity and accuracy in documenting the use and the association of prenatal caffeine exposure with birth outcomes.

## Figures and Tables

**Table 1:** Patterns of caffeine use pre- and post-pregnancy awareness (*n* = 1232).

Outcome	Trimester 1 (Pre-Pregnancy Awareness)	Trimester 1 (Post-Pregnancy Awareness)	Trimester 2	Trimester 3
Used any caffeine % (*n*)	89 (1091)	68 (834)	79 (976)	80 (980)
*Of those who used caffeine:*				
Median typical caffeine intake per consumption day (IQR)	107 (60–147)	60 (40–107)	80 (40–107)	80 (40–107)
3+ days a week % (*n*)	88 (962)	74 (616)	74 (723)	82 (802)
Median maximal caffeine intake per consumption day (IQR)	187 (120–242)	120 (80–187)	120 (83.5–187)	132 (80–200)
3+ days a week % (*n*)	27 (139)	25 (59)	22 (72)	23 (85)
Typical or maximal caffeine intake exceeds guideline % (*n*)	30 (325)	10 (82)	4 (35)	5 (50)
*Typical intake exceeds guideline (>200 mg in a day) % (n)*	16 (170)	4 (36)	8 (78)	9 (92)
*Maximal intake exceeds guideline (>200 mg in a day) % (n)*	22 (236)	7 (54)	11 (102)	13 (125)

Note: Pre-pregnancy awareness was defined as conception to the participants’ point of pregnancy awareness, and post-pregnancy awareness was defined as the point of pregnancy awareness until end of T1. IQR: inter-quartile range.

**Table 2:** Fit indices for one- to five-class latent class growth analysis models for guideline adherence (*n* = 1232).

Fit Indices	AIC	BIC	SSaBIC	Entropy	Adjusted LRT	*p*	1 Class	2 Class	3 Class	4 Class
1 class	8523.21	8538.56	8529.03	-	-	-	1.00	-	-	-
2 class	7764.07	7794.77	7775.71	0.800	730.91	<0.001	0.22	0.78	-	-
3 class	7480.06	7526.11	7497.52	0.822	277.03	<0.001	0.22	0.70	0.08	-
4 class	7407.18	7468.58	7430.46	0.871	75.35	0.113	0.11	0.05	0.74	0.10

Note: AIC: Akaike information criterion; BIC: Bayesian information criterion; SSaBIC: sample size adjusted Bayesian information criterion; LRT: Lo-Mendell-Rubin test. Note that those values italicized should be treated with caution, as parameters were fixed to avoid singularity and the model was not identified.

**Table 3:** Maternal predictors of guideline adherence trajectory group (*n* = 1232).

	Total Sample ( *n* = 1232) % (*n*)	A. Low Consumption Group (*n* = 266) % (*n*)	B. Within Guideline Group (*n* = 866) % (*n*)	C. Decreasing Heavy Use Group (*n* = 100) % (*n*)	B vs. A (ref) RRR (95% CI)	C vs. A (ref) RRR (95% CI)
**Socio-Demographics**						
**Age (at birth; M, IQR)**	33 (29–36)	32.5 (29–36)	33 (29–36)	33 (30–36)	1.01 (0.98–1.04), *p* = 0.419	1.03 (0.99–1.08), *p* = 0.161
**SES (T3)**						
Low/Moderate	36 (444)	39 (103)	36 (309)	32 (32)	-	-
High	64 (788)	61 (163)	64 (557)	68 (68)	1.14 (0.86–1.51), *p* = 0.368	1.34 (0.83–2.19), *p* = 0.236
**Country of birth (T3)**						
Australia	56 (690)	51 (136)	57 (494)	60 (60)	-	-
Other English-speaking	19 (235)	17 (46)	19 (164)	25 (25)	0.98 (0.67–1.43), *p* = 0.923	1.23 (0.69–2.19), *p* = 0.476
NESB	25 (306)	32 (84)	24 (207)	15 (15)	0.68 (0.49–0.93), *p* = 0.016	0.41 (0.22–0.76), *p* = 0.005
**Employment (T3)**						
Not in paid employment	32 (398)	32 (84)	32 (274)	40 (40)	-	-
Paid employment (including self-employ)	68 (833)	68 (182)	68 (591)	60 (60)	1.00 (0.74–1.34), *p* = 0.976	0.69 (0.43–1.12), *p* = 0.130
**University Qualification (T3)**						
No	34 (421)	30 (79)	35 (302)	40 (40)	-	-
Yes	66 (810)	70 (187)	65 (563)	60 (60)	0.79 (0.59–1.06), *p* = 0.116	0.63 (0.39–1.02), *p* = 0.062
**Living with partner (T3)**						
No	7 (90)	6 (15)	8 (65)	10 (10)	-	-
Yes	93 (1142)	94 (251)	93 (801)	90 (90)	0.74 (0.41–1.31), *p* = 0.300	0.54 (0.23–1.24), *p* = 0.146
**Pregnancy Characteristics**						
**Parity (T3)**						
0	57 (703)	67 (178)	55 (479)	46 (46)	-	-
≥1 previous children carried full-term	43 (527)	33 (88)	45 (385)	54 (54)	1.63 (1.22–2.17), *p* = 0.001	2.38 (1.49–3.80), *p* < 0.001
**Pregnancy planned (T3)**						
No	22 (273)	17 (44)	23 (199)	30 (30)	-	-
Yes	78 (958)	84 (222)	77 (666)	70 (70)	0.66 (0.46–0.95), *p* = 0.025	0.46 (0.27–0.79), *p* = 0.005
**Fertility Problems (T3)**						
No	92 (1103)	88 (227)	93 (783)	95 (93)	-	-
Yes	8 (94)	12 (30)	7 (59)	5 (5)	0.57 (0.36–0.91), *p* = 0.018	0.41 (0.15–1.08), *p* = 0.071
Substance Use						
**Tobacco use during pregnancy (T1–T3)**						
No	84 (1028)	94 (251)	82 (709)	68 (68)	-	-
Yes	17 (203)	6 (15)	18 (156)	32 (32)	3.68 (2.13–6.38), *p* < 0.001	7.88 (4.03–15.38), *p* < 0.001
**Alcohol use during pregnancy (T1–T3)**						
No	32 (391)	49 (131)	29 (247)	13 (13)	-	-
Yes	68 (841)	51 (135)	72 (619)	87 (87)	2.43 (1.83–3.22), *p* < 0.001	6.49 (3.46–12.20), *p* < 0.001
**Illicit drug use during pregnancy (T1–T3)**						
No	94 (1151)	96 (254)	94 (808)	89 (89)	-	-
Yes	6 (77)	4 (11)	6 (55)	11 (11)	1.57 (0.81–3.05), *p* = 0.181	2.85 (1.20–6.81), *p* = 0.018
**Health**						
**Pre-pregnancy body mass index (T1; M, IQR)**	22.7 (20.5–26.0)	22.5 (20.2–25.9)	22.7 (20.5–26.0)	23.6 (21.7–28.0)	1.01 (0.98–1.03), *p* = 0.598	1.06 (1.02–1.10), *p* = 0.004
**Anxiety elevated (T1)**						
No	89 (1091)	88 (233)	89 (767)	91 (91)	-	-
Yes	11 (138)	12 (33)	11 (96)	9 (9)	0.88 (0.58–1.35), *p* = 0.884	0.70 (0.32–1.52), *p* = 0.698
**Stress elevated (T1)**						
No	90 (1104)	92 (245)	90 (772)	87 (87)	-	-
Yes	10 (124)	8 (21)	10 (90)	13 (13)	1.36 (0.83–2.23), *p* = 0.224	1.74 (0.84–3.63), *p* = 0.138
**Depression elevated (T1)**						
No	81 (992)	82 (218)	81 (695)	79 (79)	-	-
Yes	19 (234)	18 (47)	19 (166)	21 (21)	1.11 (0.78–1.58), *p* = 0.575	1.23 (0.69–2.19), *p* = 0.476

Note: For body mass index, 39 participants did not self-report height and weight. T1: item assessed during interview in T1; T2: item assessed during interviewing T2; T3: item assessed in T3/reflecting entire pregnancy experience. SES: socio-economic status; NESB: non-English speaking background; M: median; IQR: inter-quartile range; T1: trimester 1; T2: trimester 2; T3: trimester 3; RRR: relative risk ratio

**Table 4:** Guideline adherence as a predictor of birth outcomes (*n* = 1232).

Models ^a^	Birth Weight (Grams) ^b^ (*n* = 1155)						SGA ^c^ (*n* = 1155)						Head Circumference (cm) ^b^ (*n* = 1012)					
	Step 1 (Unadjusted)		Step 2 (Adjusted)		Step 3 (Adjusted)		Step 1 (Unadjusted)		Step 2 (Adjusted) ^d^		Step 3 (Adjusted) ^d^		Step 1 (Unadjusted)		Step 2 (Adjusted)		Step 3 (Adjusted)	
	b (SE)	*p*	b (SE)	*p*	b (SE)	*p*	b (SE)	*p*	b (SE)	*p*	b (SE)	*p*	b (SE)	*p*	b (SE)	*p*	b (SE)	*p*
**T1 Pre-Awareness**		*R*^2^ = 0.001		*R*^2^ = 0.035		*R*^2^ = 0.083								*R*^2^ = 0.001		*R*^2^ = 0.021		*R*^2^ = 0.056
Abstinent	-	-	-	-	-	-	-	-	-	-	-	-	-	-	-	-	-	-
Within	−41.55 (49.61)	0.402	−48.93 (48.21)	0.310	−86.79 (48.63)	0.075	0.29 (0.34)	0.385	0.33 (0.33)	0.324	0.35 (0.34)	0.304	−0.14 (0.16)	0.380	−0.14 (0.16)	0.383	−0.21 (0.16)	0.186
In excess	−56.84 (53.75)	0.291	−68.58 (52.72)	0.149	−143.16 (55.95)	0.011	0.15 (0.37)	0.696	0.20 (0.37)	0.585	0.27 (0.40)	0.502	−0.11 (0.17)	0.510	−0.12 (0.17)	0.476	−0.31 (0.18)	0.089
**T1 Post-Awareness**		*R*^2^ = 0.001		*R*^2^ = 0.034		*R*^2^ = 0.080								*R*^2^ = 0.001		*R*^2^ = 0.021		*R*^2^ = 0.054
Abstinent	-	-	-	-	-	-	-	-	-	-	-	-	-	-	-	-	-	-
Within	−6.20 (33.42)	0.853	−10.25 (32.71)	0.754	−27.69 (32.93)	0.401	0.04 (0.22)	0.864	0.08 (0.21)	0.731	0.08 (0.22)	0.727	−0.08 (0.13)	0.517	−0.07 (0.13)	0.601	−0.08 (0.13)	0.517
In excess	−57.40 (70.38)	0.415	−69.55 (70.02)	0.321	−128.71 (69.04)	0.063	0.30 (0.38)	0.429	0.35 (0.38)	0.355	0.41 (0.41)	0.319	0.07 (0.24)	0.774	0.06 (0.25)	0.815	−0.08 (0.25)	0.754
**T2**		*R*^2^ = 0.003		*R*^2^ = 0.036		*R*^2^ = 0.081								*R*^2^ < 0.001		*R*^2^ = 0.021		*R*^2^ = 0.055
Abstinent	-	-	-	-	-	-	-	-	-	-	-	-	-	-	-	-	-	-
Within	56.57 (39.04)	0.148	52,96 (38.47)	0.169	23.76 (38.75)	0.540	−0.14 (0.24)	0.562	−0.11 (0.23)	0.642	−0.04 (0.25)	0.861	−0.08 (0.16)	0.596	−0.07 (0.16)	0.641	−0.13 (0.18)	0.397
In excess	−16.72 (58.80)	0.776	−28.48 (58.40)	0.626	−96.45 (60.70)	0.112	−0.09 (0.39)	0.812	−0.04 (0.39)	0.928	0.06 (0.42)	0.889	−0.11 (0.22)	0.626	−0.13 (0.22)	0.539	−0.31 (0.24)	0.169
**T3**		*R*^2^ = 0.008		*R*^2^ = 0.039		*R*^2^ = 0.080								*R*^2^ = 0.001		*R*^2^ = 0.035		*R*^2^ = 0.083
Abstinent	-	-	-	-	-	-	-	-	-	-	-	-	-	-	-	-	-	-
Within	66.17 (38.28)	0.084	63.05 (37.56)	0.094	38.70 (37.45)	0.302	−0.24 (0.23)	0.315	−0.21 (0.24)	0.362	−0.15 (0.25)	0.537	−0.03 (0.15)	0.821	0.04 (0.15)	0.813	−0.01 (0.15)	0.951
In excess	179.47 (52.13)	0.001	155.58 (52.76)	0.003	107.83 (53.20)	0.043	−0.54 (0.40)	0.177	−0.44 (0.41)	0.275	−0.37 (0.41)	0.365	0.13 (0.18)	0.472	0.11 (0.19)	0.574	0.01 (0.20)	0.977
**Trajectory Group**		*R*^2^ = 0.002		*R*^2^ = 0.035		*R*^2^ = 0.081								*R*^2^ = 0.001		*R*^2^ = 0.021		*R*^2^ = 0.056
Low Consumption group	-	-	-	-	-	-	-	-	-	-	-	-	-	-	-	-	-	-
Within Guideline group	40.84 (38.63)	0.291	34.88 (37.66)	0.354	−1.35 (38.08)	0.972	−0.02 (0.24)	0.926	0.03 (0.24)	0.907	0.09 (0.25)	0.718	−0.11 (0.15)	0.458	−0.10 (0.15)	0.53	−0.18 (0.17)	0.241
Decreasing Heavy Use group	−29.86 (65.27)	0.647	−41.28 (65.20)	0.527	−120.04 (65.11)	0.065	−0.24 (0.42)	0.580	−0.16 (0.42)	0.715	−0.04 (0.44)	0.938	−0.15 (0.21)	0.469	−0.15 (0.21)	0.471	−0.38 (0.22)	0.086

Note: ^a^ Each birth outcome model (trimester 1 (T1) pre-pregnancy awareness, T1 post-pregnancy awareness, trimester 2 (T2), trimester 3 (T3), and trajectory group) is estimated separately. Step 1 (Unadjusted) in each nested model included only the caffeine intake variables listed. Step 2 (Adjusted) also included the following mother socio-demographic and infant covariates: mother age at birth, SES (low/medium vs. high), country of birth (Australia vs. other English-speaking country vs. non-English speaking country), tertiary qualification completed (no vs. yes), living with partner (no vs. yes), and sex of child (male vs. female). Step 3 (Adjusted) also included the following variables associated with caffeine trajectory group in unadjusted multinomial regression: planned pregnancy (no vs. yes), parity (0 vs. ≥1 children carried to full-term), fertility treatment (no vs. yes), illicit drug use during pregnancy (no vs. yes), smoked tobacco during pregnancy (no vs. yes), used alcohol during pregnancy (no vs. yes), body mass index pre-pregnancy. ^b^ Linear regression analyses were conducted for these continuous outcomes (Stata nestreg regress command); coefficients and robust standard errors are presented, as well as adjusted *R*^2^. ^c^ Logistic regression analyses were conducted for these binary categorical outcomes (Stata nestreg logit command); coefficients and robust standard errors are presented. ^d^ Caution should be exacted in the interpretation of adjusted models here given lower statistical power due to few events; analyses are presented for the purposes of controlling for confounding. Small-for-gestational age (SGA): defined as birth weight less than the 10th percentile for gestational age as per Australian norms.

## Supplementary Materials

The following are available online at http://www.mdpi.com/2072-6643/10/3/319/s1, Figure S1. Proportion who do not exceed the guideline (≤200 mg; bottom panel) and whose typical/maximal intake exceeds the guideline (>200 mg; top panel) at each stage of pregnancy according to trajectory group (*n* = 1232), Figure S2. Proportion who are within the intake guideline (≤200 mg; bottom panel) and exceeding the intake guideline (>200 mg; top panel) at each stage of pregnancy according to trajectory group (using a standardized six-week point of awareness; *n* = 1232), Table S1. The number of participants excluded from the final sample and justification, Table S2. Birth outcomes according to guideline adherence within each trimester and for each trajectory group (*n* = 1232).
