# Supplementary material for: Adherence to the Caffeine Intake Guideline during Pregnancy and Birth Outcomes: A Prospective Cohort Study
Source: Nutrients. 2018 Mar 7;10(3):319. doi: 10.3390/nu10030319 (PMC5872737; doi:10.3390/nu10030319)
Supplement: Supplementary file 1 [file nutrients-10-00319-s001.zip › Supplementary/TripleB_CaffeineTrajectories_Supplementary_25012018.doc]

*Table S1*

Number of Participants Excluded from Final Sample and Justification

| **Reason for Exclusion** | **Number of Participants** |
| --- | --- |
| Gave birth to twins or triplets | 34 |
| Missing data on any of the caffeine variables | 16 |
| Pre- and post-awareness caffeine consumption in T1 was not distinguished | 77 |
| Point of pregnancy awareness was not identified | 71 |
| Immediately aware of pregnancy through IVF (and thus no pre- and post-awareness period) | 7 |
| Pregnancy awareness occurred after 13 weeks (T2), thus precluding comparison of awareness pre- and post- in T1 | 14 |
| Point of pregnancy awareness was end of T1, thus precluding comparison of awareness pre- and post- in T1 | 4 |
| Gestation occurred at 28 weeks, thus precluding any provision of T3 data | 1 |
| **Total Excluded** | **224** |

*Table S2*

Birth Outcomes According to Guideline Adherence within each Trimester and for each Trajectory Group (n=1,232)

|  | **Birthweight (grams)**  **(n=1228)**  **M (SD)** | **SGA**  **(n=1228)**  **% (n)** | **Head Circumference (cm)**  **(n=1,075)**  **M (SD)** |
| --- | --- | --- | --- |
| **T1 pre-pregnancy awareness** |  |  |  |
| Abstinent | 3471.7 (544.12) | 8.6 (12) | 34.7 (1.6) |
| Within | 3442.7 (526.6) | 10.7 (82) | 34.6 (2.0) |
| In excess | 3425.8 (494.9) | 9.6 (31) | 34.7 (1.6) |
| **T1 post-pregnancy awareness** |  |  |  |
| Abstinent | 3449.0 (541.8) | 9.6 (38) | 34.7 (2.0) |
| Within | 3442.5 (503.6) | 10.3 (77) | 34.6 (1.8) |
| In excess | 3395.9 (566.3) | 12.4 (10) | 34.8 (1.8) |
| **T2** |  |  |  |
| Abstinent | 3398.3 (545.8) | 11.4 (29) | 34.7 (2.1) |
| Within | 3460.3 (518.1) | 9.8 (85) | 34.6 (1.8) |
| In excess | 3388.4 (464.0) | 10.9 (11) | 34.7 (1.5) |
| **T3** |  |  |  |
| Abstinent | 3367.7 (526.6) | 11.9 (30) | 34.6 (1.9) |
| Within | 3448.6 (526.8) | 9.8 (83) | 34.7 (1.9) |
| In excess | 3542.4 (439.2) | 9.6 (12) | 34.7 (1.3) |
| **Trajectory Group** |  |  |  |
| Low Consumption group | 3408.3 (557.0) | 10.6 (28) | 34.7 (2.1) |
| Within Guideline group | 3457.9 (507.0) | 10.2 (88) | 34.6 (1.8) |
| Decreasing Heavy Use group | 3388.1 (529.9) | 9.1 (9) | 34.6 (1.4) |

Participants fall into one of three categories (‘abstinent’, ‘within’ or ‘in excess’ of guideline) within trimester 1 (T1) pre-pregnancy awareness, T1 post-pregnancy awareness, trimester 2 (T2), trimester 3 (T3), and trajectory group. Small-for-gestational age (SGA): defined as birth weight less than the 10^th^ percentile for gestational age as per Australian norms. M: mean; SD: standard deviation.

**Figure Legends**

*Figure S1.* Proportion who do not exceed the guideline (≤200mg; bottom panel) and whose typical/maximal intake exceeds the guideline (>200mg; top panel) at each stage of pregnancy according to trajectory group (n=1,232).

*Figure S2*. Proportion who are within the intake guideline (≤200mg; bottom panel) and exceeding the intake guideline (>200mg; top panel) at each stage of pregnancy according to trajectory group (using standardized 6-week point of awareness; n=1,232)
